# The prognostic significance of facial lymphoedema in HIV-seropositive subjects with Kaposi sarcoma

**DOI:** 10.1186/1742-6405-5-2

**Published:** 2008-01-29

**Authors:** L Feller, JN Masipa, NH Wood, EJ Raubenheimer, J Lemmer

**Affiliations:** 1 Department of Periodontology and Oral Medicine, University of Limpopo School of Dentistry, Pretoria, South Africa; 2 Department of Maxillofacial and Oral Surgery, University of Limpopo School of Dentistry, Pretoria, South Africa; 3 Department of Oral Pathology, University of Limpopo School of Dentistry, Pretoria, South Africa

## Abstract

**Background:**

Kaposi Sarcoma (KS) is a multifocal angioproliferative neoplasm characterized by inflammation, oedema, neoangiogenesis and spindle cell proliferation. The pathogenesis of human immunodeficiency virus (HIV)-associated KS (HIV-KS) is multifactorial. HHV-8 is an essential factor but not in itself sufficient to cause HIV-KS, the development of which is influenced by HIV, by increased production of cytokines and by growth factors. Whether HIV-KS is a true malignancy or a reactive hyperplastic inflammatory condition is debatable.

**Results and Conclusion:**

Oedema of the face, legs and hands is a prominent feature of HIV-KS and is probably caused by lymphoedema related to the HIV-KS lesions. The cases of two HIV-seropositive subjects with KS-associated facial lymphoedema are reported. Extensive oral HIV-KS in association with facial oedema in the absence of anti-retroviral treatment appears to be an indication of a poor prognosis.

## Introduction

Kaposi sarcoma (KS) is an endothelial tumour of lymphatic endothelial origin that affects mucocutanous sites, but may also involve internal organs [1]. Human immunodeficiency virus (HIV)-associated KS (HIV-KS) is the most common tumour in HIV infection and it may occur at any level of CD4+ T cell count during HIV infection, but usually affects HIV-seropositive subjects with CD4+ T cell count below 200 cells/μl [2].

The natural course of HIV-KS is unpredictable. It may be either a mild or a life-threatening disease but the overall prognosis without treatment is poor. Aggressive HIV-KS is associated with extensive intraoral exophytic lesions, sometimes with oedema and sometimes as in the case of aggressive HIV-KS at any site, with pulmonary involvement [3-5].

Lymphoedema associated with HIV-KS commonly affects the face, the neck, the external genitalia and the lower extremities. It may be present before the appearance of clinically overt KS lesions and the lymphoedematous site is predisposed to secondary bacterial infections [6].

Oral HIV-KS lesions are single or multifocal, initially present as macules that may progress to papulo-nodular lesions and eventually become confluent to form large exophytic masses [7]. The lesions are bluish-purple or red, may be indolent or may be locally aggressive [8,9]. Oral HIV-KS most frequently affects the hard palate, the gingivae and the dorsum of the tongue, and in advanced cases the tumour may progress into the underlying bone [10]. In 71% of HIV-seropositive subjects with KS the oral cavity will be affected at some time during their KS disease, and subjects with oral HIV-KS have a higher mortality rate than subjects with exclusively cutaneous manifestations [3,11].

The aim of this paper is to report that the onset of facial lymphoedema in two subjects with extensive HIV-KS of the mouth of some duration who had not received antiretroviral treatment was rapidly followed by death. We suggest that the onset of facial lymphoedema under circumstances comparable to those mentioned above, may have serious prognosticatory significance. As far as we are aware this observation has not previously been reported.

## Case presentation

### Case 1

**Figure 1:**
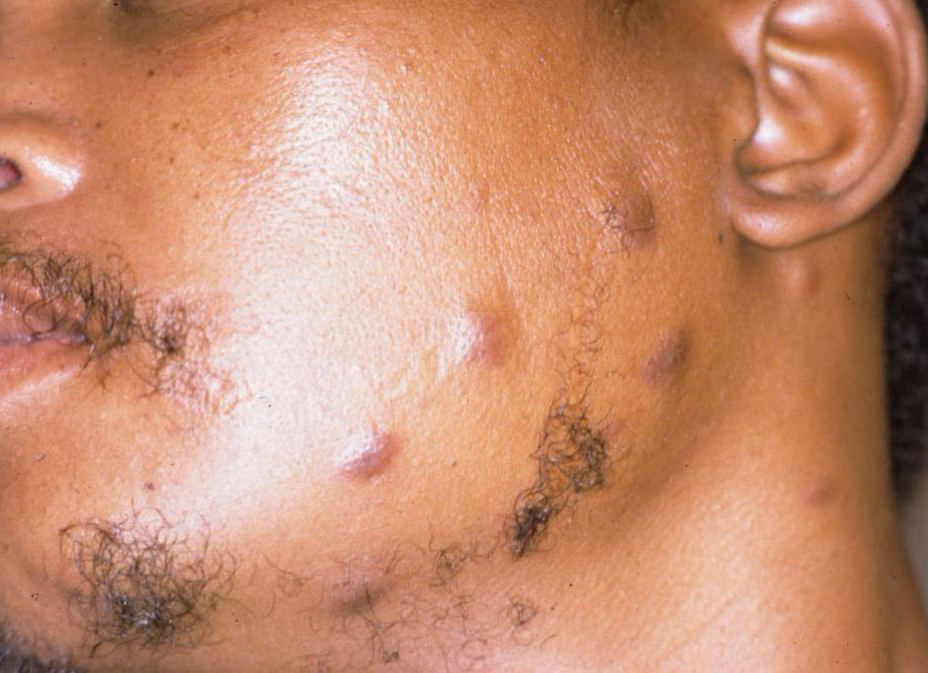
KS nodules on the face at initial presentation.

**Figure 2:**
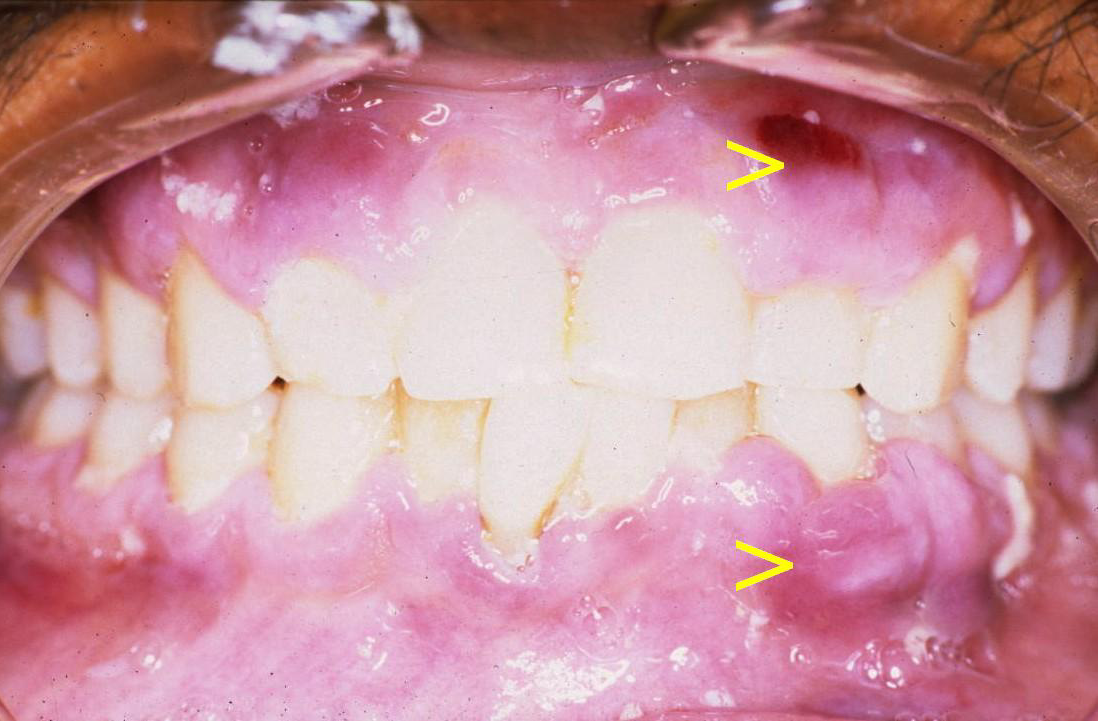
Multifocal maculo-papular KS lesions on the gingivae.

**Figure 3:**
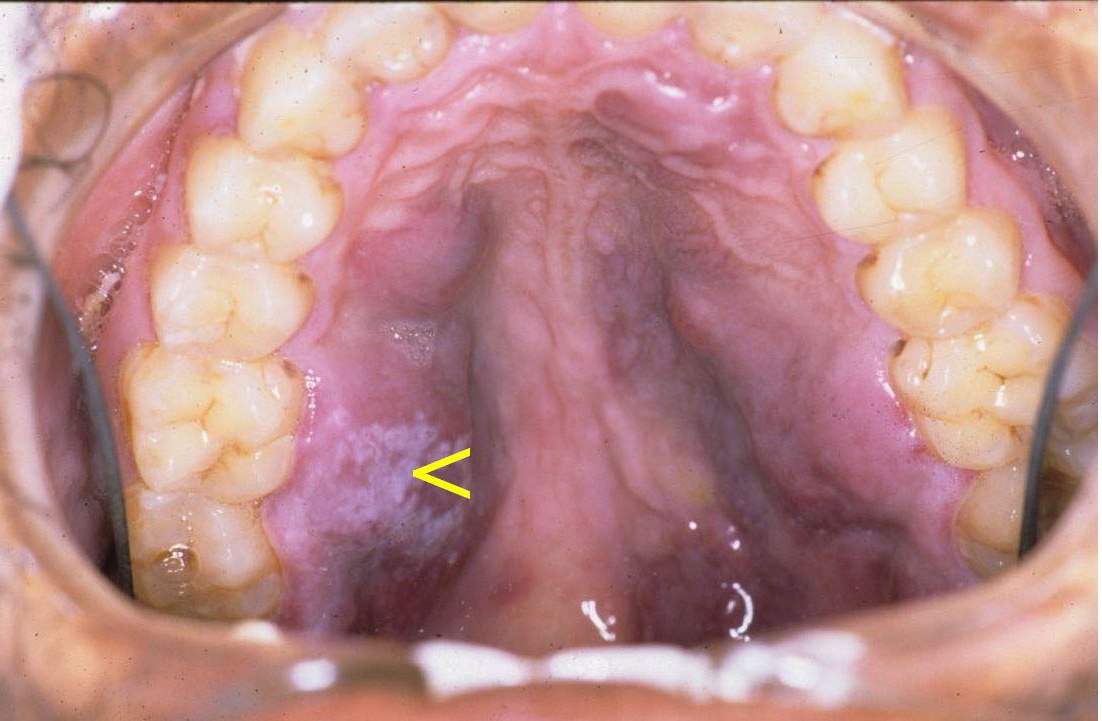
Multifocal purple-red maculo-papular KS lesions on the palate. Note the pseudomembranous candidiasis (arrow).

A 28-year-old black male with an unremarkable medical history presented with numerous nodules on the face (Fig 1), and with multifocal, purple-red, maculo-papular lesions on the gingivae (Fig 2), and on the hard palate (Fig 3). The patient reported that the facial and intra-oral lesions had appeared concurrently three months prior to our examination.

KS was provisionally diagnosed. Serological tests confirmed HIV infection, and microscopic examination of a biopsy specimen from the palate showed pronounced vascular and spindle cell proliferation, slit-like vascular spaces with extravasated red blood cells. These findings provided confirmation of the provisional diagnosis of KS.

**Figure 4:**
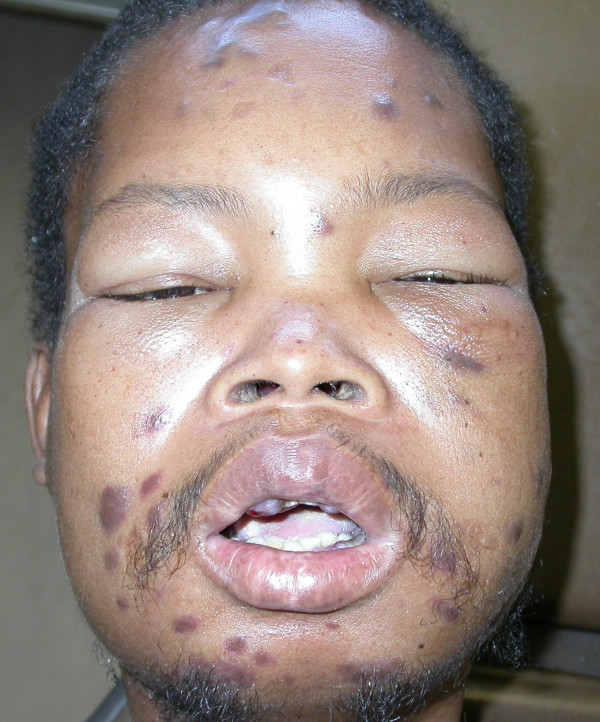
Photograph of the face three months after the initial presentation (Fig 1). Note the oedema of the face, in particular of the periorbital tissues.

**Figure 5:**
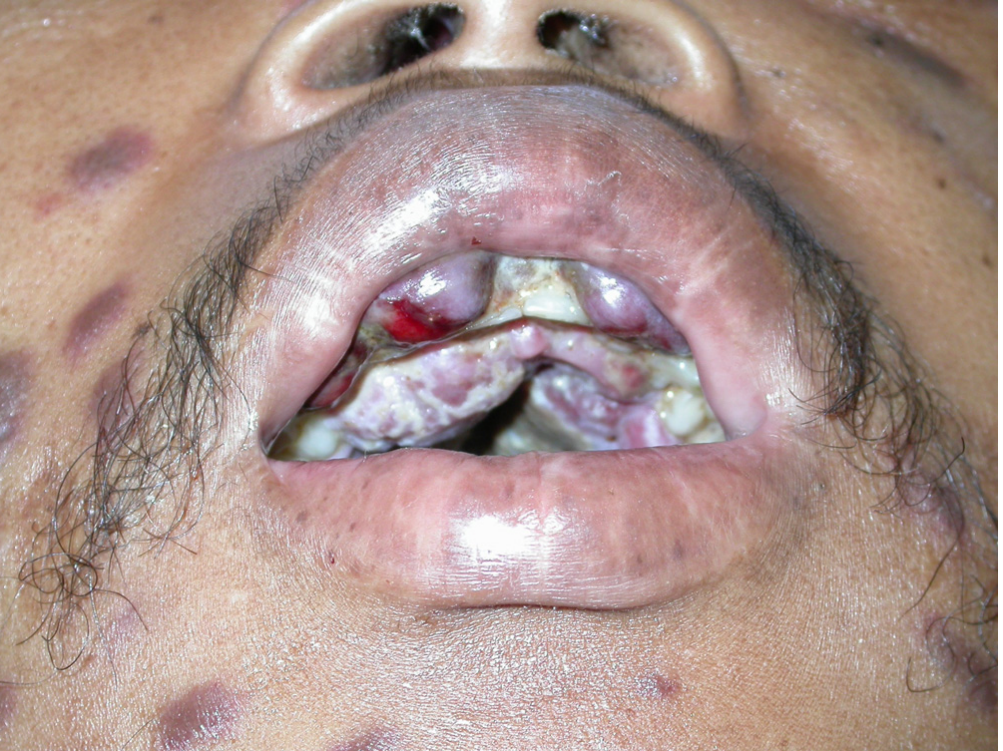
Photograph showing the worsening of the palatal lesions (three months after the initial examination (Figs 2 & 3).

The patient refused anti-retroviral treatment and systemic cytotoxic chemotherapy, and was temporarily lost to follow-up but returned to our clinic three months later. He now presented with severe oedema of the face, an increase in number of the facial KS lesions (Fig 4), and extensive enlargement of the oral KS lesions affecting the maxillary and mandibular gingivae and the hard palate. (Fig 5). The patient was in severe pain, could not eat, and had difficulty speaking. He again refused any investigations and treatment, returned home and died two weeks later, the cause of death being undetermined.

### Case 2

**Figure 6:**
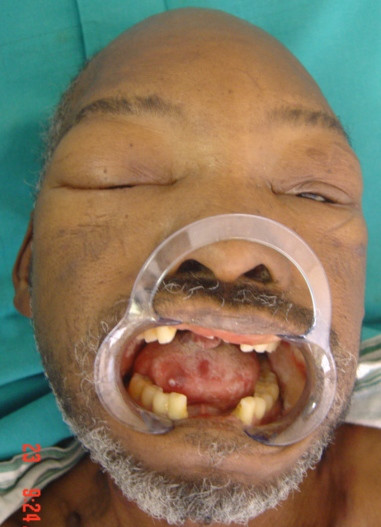
Oedema of the face. Note the pronounced periorbital oedema.

A 55-year old HIV-seropositive man with a CD4+ T cell count of 12 × 10^6^/l and CD4+ T cell and a percentage of total lymphocytes of 2.11%, had extensive cutaneous and oral lesions as well as pronounced oedema of the face (Fig 6), and the lower extremities.

The patient was wasted, had been treated for tuberculosis but had chronic obstructive lung disease, pulmonary hypertension with right ventricular enlargement, and intractable gastroenteritis. He had developed several cutaneous Kaposi sarcomata two months prior to our examination. The patient reported that the facial oedema and the oral lesions had been rapidly getting worse over the last three weeks.

**Figure 7:**
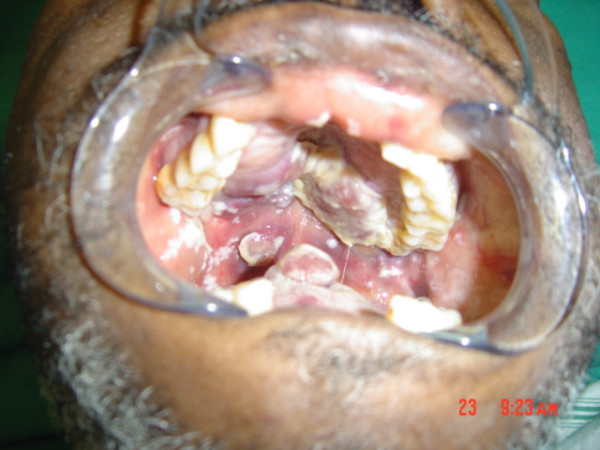
Intraoral view showing extensive KS lesions on the hard palate, the soft palate and the dorsum of the tongue.

The oral lesions were exophytic, red lobulated masses affecting the palate, maxillary gingivae and the dorsum of the tongue (Fig 7). Microscopic examination of a biopsy specimen from the tongue showed features consistent with those of KS.

The patient died 7 days after our examination, while systemic investigation was in progress, and before cytotoxic chemotherapy could be initiated.

## Discussion

### Lymphoedema

Lymphoedema is characterized by an abnormal accumulation of protein-rich interstitial fluid in the presence of normal capillary filtration, resulting in swelling of the affected soft tissue. This is in contrast to oedema that develops when capillary filtration rate exceeds lymph drainage [12].

Lymphoedema can be categorized as primary and secondary. Primary lymphoedema is rare and is associated with agenesis, hypoplasia or ectasia of lymphatic channels. Secondary lymphoedema usually occurs due to damage to, or obstruction of normal lymphatic channels interfering with lymphatic flow. Common causes of secondary lymphoedema include infections that lead to obliteration of lymphatic lumina, extensive surgical excision of lymph nodes, post surgical scarring, malignancies that start in, or spread to lymph nodes, and radiation treatment that causes fibrosis, blocking the lymphatics. Lymphoedema affects mainly the extremities, which have limited collateral lymphatics which cannot readily drain excess fluid [12,13].

Chronic damage to, or blockade of lymphatic channels may result in low-output failure of lymph circulation. Long-standing, high-protein lymphoedema has the potential to stimulate angiogenesis and neovascularization both of lymphatics and of blood vessels, lipid deposition, subcutaneous fibrosis and hyperkeratosis. In addition, stasis of lymph implies failure of transport of fluid, crystalloids, macro-molecules and lymphoid cells through the lymphatics, the lymph nodes, and from the tissues back to the blood stream. There will also be local immune impairment and depletion of nutritional factors in the affected lymphoedemateous regions [12,14]. The development of lymphoedema may be attributed to the high interstitial protein concentration with increased osmotic pressure that causes retention of fluid in the connective tissue [15], and to release of growth factors, in particular vascular endothelial growth factor and basic fibroblast growth factor, that may bring about subcutaneous fibrosis and hyperkeratosis [15,16].

### The local environment in chronic lymphoedema

Lymphoedema is associated with local immune impairment. Migration of immunoregulatory cells such as dendritic cells, T lymphocytes and macrophages from peripheral tissue sites to regional lymph nodes via lymphatic vessels is compromised in the setting of lymphoedema. This results in ineffective clearance of antigens and in impaired local adaptive cellular immune responses as well as in immune surveillance. In addition, in lymphoedemateous areas there are alterations in the profiles of the local cytokines, growth factors and adhesion molecules and there is dysregulation of immunoinflammatory reactions leading to progressive endothelial cell proliferation with the development of abnormalities in the pattern of the lymphatic vessels. These alterations in the local microenvironment and in the immune competence of the lymphoedemateous areas may play roles of cofactors in the initiation and promotion of KS [12,17].

### Lymphoedema associated with HIV-KS

Lymphoedema may precede the development of HIV-KS; may be present at the time of diagnosis of HIV-KS; or may develop later in parallel with the progression of HIV-KS disease [17].

The clinical oedema observed in some subjects with KS may be pronounced, as in the presented cases, and affects mainly the lower extremities and the periorbital areas [6,15]. Lymphangioscintigraphy in subjects with KS shows a variety of abnormal patterns of the lymphatic channels, corresponding to the distribution of the cutaneous KS lesions [18], and to the clinical lymphoedema. At times the oedema with its associated subcutaneous fibrosis and with hyperkeratosis may camouflage the cutaneous KS lesions and make the diagnosis of KS difficult [15,19].

In addition to the factors previously, mentioned HHV-8 infection of lymphatic endothelial cells and of cells resident in lymph nodes may cause both damage to lymphatic channels and enlargement of lymph nodes, with impeded lymphatic drainage and consequent lymphoedema [12,15,18]. HHV-8 is a necessary factor but not in its own sufficient to cause KS: but chronic lymphoedema together with HHV-8 may initiate the development of in-situ KS that with time progresses to become clinically manifest KS [20].

Our first patient refused hospitalization or any further investigations or treatment at all, therefore we can only state that the facial oedema was present but not whether it may have had a non-HIV/KS related cause. The second patient was in hospital in the care of physicians who recorded none of the other possible causes of facial lymphoedema than HIV-KS.

Lymphoedema that precedes KS may in the presence of HHV-8 infection be a predisposing factor to the development of KS from lymphatic endothelial cells [16,20]. On the other hand, it has been shown [19,20], that fibroma-like nodules in the submucosa, of uncertain etiology, in the presence of lymphoedema and HHV-8 may evolve to in-situ KS.

The simultaneous development of lymphoedema and KS, or the development of lymphoedema in parallel with the progression of established KS may be attributed to the HHV-8-induced exuberant proliferation of endothelial cells that may lead to the occlusion of lymphatic vascular lumens. When this is widespread and involves multiple lymphatic channels, it has the potential to cause lymphoedema. The obliteration of the lymphatic channels may be supplemented by their compression by the enlarging KS [18,19]. This process is most probably responsible for the severe clinical oedema associated with rapidly progressive HIV-KS disease.

### Treatment of HIV-KS associated lymphoedema

Treatment of lymphoedema associated with HIV-KS focuses on treatment of both the HIV-KS and the lymphoedema. Highly active antiretroviral therapy (HAART) together with cytotoxic chemotherapy is the treatment of choice for the management of HIV-KS. At times this may also improve the associated lymphoedema. The lymphoedema should be treated in parallel to the treatment of HIV-KS with diuretic agents, and in cases where the lower extremities are involved, by elevation of the limbs and compressive dressings. The preservation of skin integrity is important to prevent secondary bacterial and fungal infections. Application of emollients to the affected lymphoedematous skin may prevent cutaneous breakdown. If breakdown does occur, application of topical antimicrobial agents is necessary [21].

### Comments

According to the AIDS clinical trial group staging classification for AIDS-associated Kaposi sarcoma, lymphoedema and exophytic HIV-KS oral lesions are independently associated with poor prognosis [3,4]. The two cases reported here demonstrate that in HIV-seropositive subjects with established nodular oral KS lesions, the development of facial lymphoedema rapidly leads to death. The pathogenic mechanisms that bring about the rapid development of facial lymphoedema and that lead to death are obscure.

In sub-Saharan Africa, the natural course of HIV-KS in the absence of HAART is characterized by rapid disease progression associated with high HHV-8 burden and short life expectancy. In this geographic region, HIV-KS has reached epidemic proportions [22,23]. HIV-KS in the mouth is common, and subjects with extensive exophytic oral lesions and tumour-associated oedema have higher death rates than HIV-seropositive subjects having exclusively cutaneous lesions [3].

Since there is no cure for HIV-KS, conventionally its treatment focused on control of tumour growth and palliation. HAART should always be instituted in HAART-naïve HIV-seropositive subjects with KS, since it promotes regression of KS lesions. However, in about 6,5% of these subjects HIV-KS may flare up shortly after the introduction of HAART as an immune reconstitution inflammatory syndrome (IRIS) [24].

In light of the cases presented here and our personal experience, it might be advisable to treat oral HIV-KS with cytotoxic chemotherapy at early maculo-papular stages when the lesions are still asymptomatic. Such a treatment protocol might have several advantages. Firstly, systemic cytotoxic chemotherapy might prevent or at least delay the development of extensive exophytic oral lesions that have a poor prognosis; secondly it might prevent the development of lymphoedema associated with late stages of HIV-KS; and thirdly, in the early stages of oral HIV-KS, limited cytotoxic chemotherapy is sufficient to control tumour growth compared to more extensive chemotherapy regimen needed to treat advanced oral HIV-KS. Indeed, systemic chemotherapy given to HIV-seropositive subjects has the risk of exaggerating the existing state of immuno-suppression, thus further increasing the susceptibility to opportunistic infections and neoplasms, however, limited doses of cytotoxic chemotherapy is not usually associated with such adverse effects.
